# Reliability of functional gait assessment in patients with Parkinson disease: Interrater and intrarater reliability and internal consistency

**DOI:** 10.1097/MD.0000000000004545

**Published:** 2016-08-26

**Authors:** Yaqin Yang, Yongjun Wang, Yanan Zhou, Chen Chen, Deli Xing

**Affiliations:** Department of Neurology, Beijing Tiantan Hospital, Capital Medical University, Beijing, China.

**Keywords:** assessment, balance, gait, Parkinson disease, reliability

## Abstract

To determine the reliability of the functional gait assessment (FGA) as a measure of balance and gait in patients with Parkinson disease (PD).

This study involved121 inpatients with PD (mean age 61.9 years). The participants were scored using the FGA by 2 raters, and the testing procedure was videotaped. One of the raters re-assessed the same FGA test via review of the videotaped test 4 weeks later.

The interrater and intrarater reliability of the total FGA score was found to be excellent, with an intraclass correlation coefficient of 0.99. The reliability of single items of the FGA ranged from 0.49 to 0.99. The internal consistency of the FGA scores was 0.94.

The FGA has high inter-rater and intrarater reliability, and internal consistency for evaluating balance and gait disorders in patients with PD.

## 1 Introduction

Parkinson disease (PD) is a common neurodegenerative disease that is typically manifested clinically by dyskinesia, including tremors, muscle rigidity, bradykinesia, and gait abnormalities. Postural instability and balance problems are troublesome symptoms for patients with PD, as they seriously affect quality of life and may lead to falls and a subsequent increase in morbidity and mortality.^[1–4]^ Many clinical scales have been developed to objectively evaluate balance and gait disorders. The functional gait assessment (FGA) proposed by Wrisley et al^[5]^ in 2004 is one such scale used to measure disturbances in balance and gait.

The FGA was developed as a modified version of the Dynamic Gait Index (DGI).^[6]^ The DGI has been used for several years to assess postural stability during gait tasks in adults over 60 years of age.^[7–10]^ However, some researchers discovered that the DGI was not sensitive when used for patients with walking impairments.^[11,12]^ Additionally, the instructions for the DGI are ambiguous for several items, leading to difficultly in scoring by raters. To address these problems, Wrisley et al^[5]^ created the FGA by modifying the DGI. The FGA is a 10-item gait test that includes 7 of the 8 items from the original DGI (item 7, “walking around obstacles,” was of insufficient difficulty to be included) and adds 3 new items. The 10 items of the FGA are as follows: gait on a level surface; change in gait speed; gait with horizontal head turns; gait with vertical head turns; gait and pivot turn; step over obstacle; gait with narrow base of support; gait with eyes closed; ambulating backwards; and steps. Each item is scored on a 4-pointordinal scale with scores of 0, 1, 2, and 3. The maximum total score is 30; higher scores represent better balance and gait ability.^[4,5]^

The reliability of a new scale should be tested in different people. Scale reliability refers to the degree of approximation of repeated measurements under the same conditions. It is used to evaluate scale stability and consistency, and may vary over time and between subjects. Reliability encompasses both external and internal reliability. The former includes intrarater and inter-rater reliability, whereas the latter refers to the consistency of the elements in the scale's items.

Since the FGA has been published, several researchers have analyzed its reliability in patients with different diseases and disorders. Wrisley et al^[5]^ tested the reliability of the FGA in patients with vestibular disorders. The inter-rater and intrarater reliability of the total FGA score were found to be high, with intraclass correlation coefficients (ICCs) of 0.84 and 0.83, respectively.^[5]^ The internal consistency of the FGA was determined to be 0.79.^[5]^ Walker et al^[13]^ published reference-group data for the FGA by stratifying people aged 40 to 89 years into decade cohorts. The ICC for inter-rater reliability was 0.93 in their study. The mean FGA scores systematically decreased with increasing age, and ranged from 29/30 for adults in their 40s to 21/30 for adults in their 80s. At the same time, there was an increase in the standard deviation of the total scores with each decade, demonstrating that the variability of the performance on the FGA increased with age, which is in agreement with the findings of other research studies on gait and balance in old adults.^[14–16]^ Thieme et al^[17]^ tested the reliability and validity of the FGA in patients with subacute stroke; the intrarater and inter-rater reliability of total FGA scores were found to be excellent, with ICCs of 0.97 and 0.94, respectively. Leddy et al^[18]^ compared the Berg Balance Scale (BBS), FGA, and a newly developed Balance Evaluation Systems Test (BESTest) in community-dwelling patients with PD. The inter-rater reliability was excellent for all 3 tests, with ICCs greater than 0.93.

Since the FGA was developed, its reliability has been assessed in subjects with vestibular dysfunction, community-dwelling elders, and stroke patients.^[5,13,17]^ Leddy et al^[18]^ reported the reliability of the FGA in community-dwelling patients with PD. The purpose of the present study was to evaluate balance and gait disorders in hospitalized patients with PD to further verify the reliability of the FGA. We predicted that the FGA would be a reliable means of assessing balance and gait in patients with PD, based on previous research. This study may provide tools for the clinical assessment and rehabilitation of patients with PD, and facilitate their motor-function training.

## 2 Materials and methods

### 2.1 Participants

All inpatients with PD who were hospitalized in the Movement Impairment Ward of the Department of Neurology at Beijing Tiantan Hospital between March 2011 and December 2011 were screened. In all, 121 in-patients (82 males and 39 females), representing 28.3% of the 428 patients who were screened, were enrolled in this study. The primary reason for hospitalization was to optimize PD treatment (medication adjustments). The anti-Parkinson medicines administered included compound levodopa (levodopa with benserazide or carbidopa), dopamine receptor agonists (piribedil and pramipexole), catechol-*O*-methyltransferase inhibitor (entacapone), amantadine, and antimuscarinic drugs (Artane).

All participants met the following inclusion criteria: diagnosed with idiopathic PD according to the diagnostic criteria of the UK Parkinson Disease Society Brain Bank,^[19]^ able to stand still without support for at least 1 minute, and Mini-Mental State Examination score ≥24. Subjects with the following criteria were excluded: diagnosed with secondary Parkinson syndrome or Parkinson plus syndrome, inability to walk at least 10 m without physical assistance or walking aids (no participant was allowed the use of a walking assist device), or presence of a comorbidity affecting motor function (such as stroke, amputation, or visual impairment).^[4]^

### 2.2 Ethics statement

This study was approved by the Medical Ethics Committee of Beijing Tiantan Hospital, in compliance with the Declaration of Helsinki. All participants or their legal representatives signed informed consent forms from the Medical Ethics Committee of Beijing Tiantan Hospital.

### 2.3 Procedure

Patient characteristics were recorded upon admission. The following variables were extracted from the participants’ medical records: age, sex, duration of PD (in years), medical and surgical history, current medication regimen, and modified Hoehn and Yahr (HY) scale.^[20]^ The HY scale is used to evaluate disease severity and duration (higher scores indicating worse impairment).^[20]^ FGA was conducted in the rehabilitation room of the Department of Neurology at Beijing Tiantan Hospital. All participants underwent FGAs performed by 2 designated licensed physical therapists (rater A and rater B) at the same time. Both raters had received FGA training and practiced the FGA on 2 healthy adults and 2 patients with PD. The assessments were completed within 1 day, 24 to 48 hours after hospital admission. The FGA was conducted in the ON medication phase (approximately 1 hour after taking anti-PD medications). The evaluation time was 10 to 30 minutes.^[4]^

The 2 raters scored the performance on the FGA by directly observing the patients. They were instructed not to discuss the grading criteria or the test with each other during the entire sequence, and were blinded to each other's results. Additionally, the FGA procedure was video-recorded. Rater A assessed the performances of the same FGA test at separate time points via review of the videotaped recording. There was a minimum interval of 4 weeks between the 2 ratings to minimize possible memory effects.

### 2.4 Data analysis

All analyses were performed using SPSS version 17.0 (SPSS Inc., Chicago, IL). For sample characteristics, descriptive statistics were used. The inter-rater and intrarater reliability of the total FGA score were statistically evaluated using ICCs and a 2-way random-effects model. Inter-rater reliability was calculated using the 2 raters’ scores obtained after direct observation of the FGA test. Intrarater reliability was calculated using the 2 ratings of rater A obtained after direct and videotaped observation. The reliability of single items was calculated using the weighted kappa statistic. Agreement strengths for kappa values were classified as follows: <0, poor; 0.00 to 0.20, slight; 0.21 to 0.40, fair; 0.41 to 0.60, moderate; 0.61 to 0.80, substantial; and 0.81 to 1.00, almost perfect.^[21]^ Internal consistency or the homogeneity of the FGA was determined using Cronbach alpha. A Cronbach alpha value ≥0.80 indicated good internal consistency.^[22]^ The correlation coefficient of 1 item with the total score of the remaining items was denoted by the “corrected item-total correlation.” A coefficient less than 0.40 indicated a low degree of correlation between the item and the remaining items.^[22]^

## 3 Results

A total of 121 inpatients (82 males and 39 females) completed the study. The baseline patient characteristics, FGA scores, and HY stages are shown in Table 1.

**Table 1:**
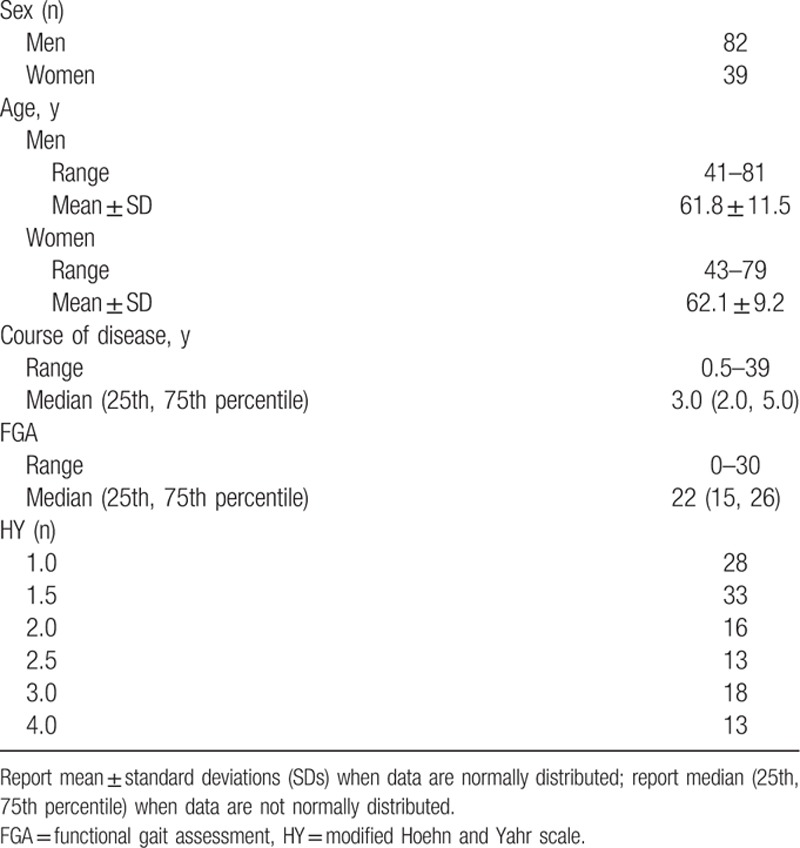
Participant demographics (n = 121).

### 3.1 Inter-rater and intrarater reliability

The results for inter-rater and intrarater reliability are shown in Table 2. Inter-rater reliability was found to be excellent, with an ICC for the total score of 0.99 (95% confidence interval [CI] 0.99–1.00). Kappa values for single items ranged from 0.49 (item 3) to 0.98 (item 8). Kappa values for items 3 and 4 were fair at 0.49 and 0.60, respectively, and those for the other items were substantial or almost perfect.^[21]^

**Table 2:**
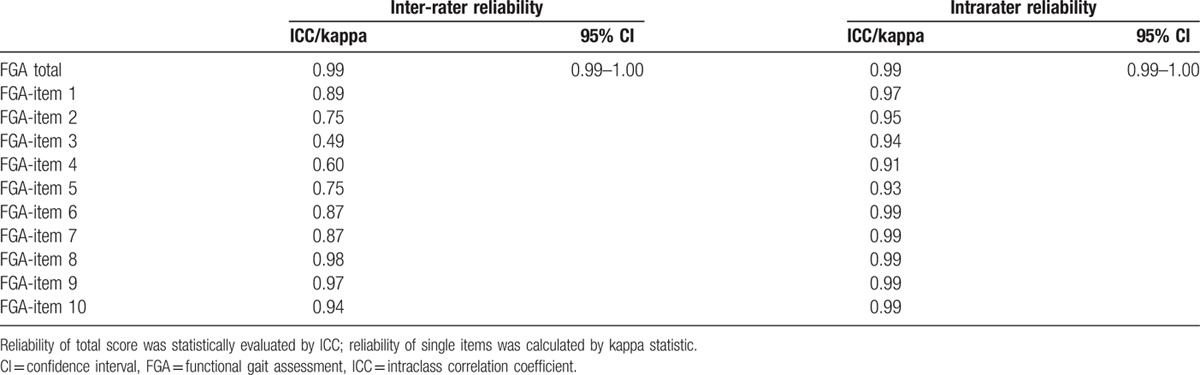
Inter-rater and intrarater reliability of FGA.

Excellent intrarater reliability was observed for the total FGA score, with an ICC of 0.99 (95% CI 0.99–1.00). For single items, kappa values ranged from 0.91 (item 4) to 0.99 (items 6–10). Intrarater reliability for all items was almost perfect.^[21]^

### 3.2 Internal consistency

The Cronbach alpha value for the total FGA score was 0.94. The corrected item-total correlations ranged from 0.65 to 0.80. Each item of the FGA was omitted in turn, and the alpha value of the remaining items was calculated; the alpha value thus calculated was found to be 0.93 in all cases (Table 3).

**Table 3:**
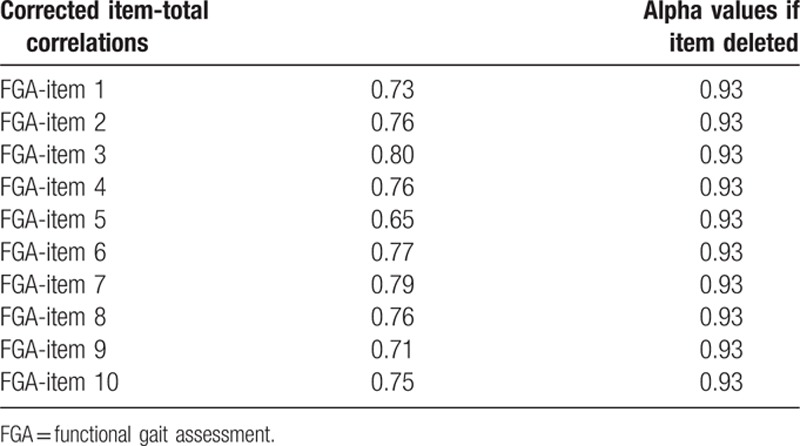
Internal consistency of FGA.

## 4 Discussion

Intrarater and inter-rater reliability represents the external reliability of a scale, and refers to the consistency of measurements obtained at different times or by different evaluators, respectively. When the FGA was used to assess hospitalized patients with PD, the inter-rater reliability of the whole scale was found to be excellent, with an ICC of 0.99. This value is higher than those previously reported for FGA reliability.^[5,13,17,18]^ In the study on patients with vestibular dysfunction by Wrisley et al,^[5]^ the raters were given just 10 minues to familiarize themselves with the FGA items, scoring rules, and guidance, and the inter-rater reliability was 0.84. In several other studies, the raters received prior training, and the inter-rater reliability was markedly higher (0.93–0.94), and was similar to the results of the present study.^[13,17,18]^ These observations suggest that adequate training of raters on how to use the FGA scale improves evaluation reliability.

In the present study, the inter-rater reliability of a single item on the FGA ranged from 0.49 to 0.98. Low inter-rater reliability was observed for items 3 (gait with horizontal head turns) and 4 (gait with vertical head turns), with ICCs of 0.49 and 0.60, respectively. The other items showed high inter-rater reliability. The inter-rater differences for items 3 and 4 were mainly focused on scores 3 and 2, which referred to “Performs head turns smoothly with no change in gait” and “Performs head turns smoothly with slight change in gait velocity (e.g., minor disruption to smooth gait path),” respectively. It is likely that bradykinesia and walking slowly were the main clinical manifestations in PD patients, some of whom may have exhibited freezing behaviors. Therefore, slow walking and walking pauses were common manifestations. The difficulty in clearly defining the boundaries between no change and slight change in gait velocity likely led to the scoring discrepancies.

In the present study, the whole scale and individual FGA items had excellent intrarater reliability. The ICC of the full FGA was 0.99, and the kappa values of individual items ranged from 0.91 to 0.99, which are higher than the values previously reported in patients with vestibular dysfunction, stroke, and PD.^[5,17,18]^ In the vestibular dysfunction study, intrarater reliability with a 2-hour interval was just 0.83,^[5]^ whereas secondary evaluation of video data from the first evaluation of stroke patients yielded an intrarater reliability of 0.97,^[17]^ which is similar to that in the present study. In the study by Leddy et al^[18]^ on patients with PD, 2 evaluations were performed 2 weeks apart. Even though the evaluations were performed at the same time of day to try to ensure that the patients were in similar states, intrarater reliability showed considerable fluctuation.^[18]^ The functional status of PD patients is affected by multiple factors, including anti-PD drugs, physiological function, mood, and diet, and patients may show significant differences in balance and gait between the “ON” and “OFF” periods of drugs. In the present study, videotaped data were used to overcome the abovementioned disadvantages. The above findings suggest that appropriate method selection is important for FGA intrarater reliability.

Internal reliability refers to the consistency of the elements in a scale's items, and is usually expressed as the Cronbach alpha value, with higher values signifying greater internal reliability. “Corrected item-total correlation” is an indicator used to judge if the homogeneity of the latent trait of the items to be measured (characteristics of balance and gait in the present study) is identical to that of the remaining items. After a certain item is omitted, the alpha of the total score of the remaining items should become smaller than the Cronbach alpha of the total scale. If the alpha of the scale becomes larger after a certain item is deleted, then the homogeneity of the latent trait of the item to be measured is not identical to that of the other items.

In the present study, the Cronbach alpha for the total FGA score was 0.94, which was higher than that reported by Wrisley et al^[5]^ in patients with vestibular dysfunction. This indicates that the internal consistency of the FGA was ideal when applied to patients with PD. The “corrected item-total correlation” ranged from 0.65 to 0.80, which is >0.40, suggesting that there was good correlation between each item and the remaining items, and that the elements of the measurements were consistent. Of these, item 5 (gait and pivot turn) had the lowest correlation with the other items (*r* = 0.65). This item required the patients to stand and turn around while they walked, and the score was based on the time necessary to perform this action. Due to marked bradykinesia, patients with PD have difficulty turning over, standing, sitting, and turning around, leading to the phenomenon of freezing gait. Item 5 assessed a major gait problem experienced by patients with PD. In contrast, the other items focused on common actions related to balance and walking; therefore, they might not reflect the gait disorder, and also item 5 in PD patients. Consequently, item 5 weakly correlated with the other items. All alpha values calculated after the omission of a single item were 0.93. Because this value is <0.94, it indicates that the same concept was measured by every single item and the remaining items, namely, balance and gait disorder.

### 4.1 Limitations

The present study population comprised PD patients who were hospitalized at a single center. It is uncertain whether the same conclusions would be reached if the FGA was applied to other PD populations, such as community-dwelling individuals. The use of walking-assist devices is allowed in the FGA, but no participant was allowed to use them in this study. This means that more patients with relatively serious conditions were not included in this study, which could affect the scale reliability. In the present study, the 2 raters were female physical therapists who were working in the Department of Neurology, had graduated from medical school within the past 2 years, and had similar educational backgrounds and practical experience. It is possible that the reliability may change if the raters have different backgrounds.

## 5 Conclusions

The FGA has high external and internal reliability with good scale stability for evaluating balance and gait disorders in patients with PD. To improve its clinical utility, further studies should assess the concurrent validity, discriminative validity, and predictive validity of falls, and also the responsiveness of the FGA in patients with PD.

## Acknowledgment

We thank Hui Chen, PhD, for her assistance and advice on statistics during the study process.
